# GADD45B as a Prognostic and Predictive Biomarker in Stage II Colorectal Cancer

**DOI:** 10.3390/genes9070361

**Published:** 2018-07-19

**Authors:** Zhixun Zhao, Yibo Gao, Xu Guan, Zheng Liu, Zheng Jiang, Xiuyun Liu, Huixin Lin, Ming Yang, Chunxiang Li, Runkun Yang, Shuangmei Zou, Xishan Wang

**Affiliations:** 1 Department of Colorectal Surgery, The Second Affiliated Hospital of Harbin Medical University, Harbin 150086, China; xunnna@126.com (Z.Z.); gaoyibo@cicams.ac.cn (Y.G.); yrkun0728@126.com (R.Y.); 2 Department of Thoracic Surgery, National Cancer Center/National Clinical Research Center for Cancer/Cancer Hospital, Chinese Academy of Medical Sciences and Peking Union Medical College, Beijing 100020, China; lichunxiang@cicams.ac.cn; 3 Department of Colorectal Surgery, National Cancer Center/National Clinical Research Center for Cancer/Cancer Hospital, Chinese Academy of Medical Sciences and Peking Union Medical College, Beijing 100020, China; drguanxu@163.com (X.G.); zheng.liu@yale.edu (Z.L.); zone-j@hotmail.com (Z.J.); yangming_0517@163.com (M.Y.); 4 Department of Pathology, National Cancer Center/National Clinical Research Center for Cancer/Cancer Hospital, Chinese Academy of Medical Sciences and Peking Union Medical College, Beijing 100020, China; 13366818618@126.com; 5 Geneis (Beijing) Co., Ltd., Beijing 100102, China; linhx2@geneis.cn

**Keywords:** colorectal cancer, the growth arrest DNA damage-inducible 45 beta (*GADD45B*), prognosis, stage II, biomarkers, adjuvant chemotherapy

## Abstract

*GADD45B* acts as a member of the growth arrest DNA damage-inducible gene family, which has demonstrated to play critical roles in DNA damage repair, cell growth, and apoptosis. This study aimed to explore the potential relationship between *GADD45B* expression and tumor progression and evaluate the clinical value of *GADD45B* in stage II colorectal cancer (CRC). The expression patterns and prognostic value of *GADD45B* in CRC were analyzed based on The Cancer Genomic Atlas (TCGA). *GADD45B* expression features of 306 patients with stage II CRC and 201 patients with liver metastasis of CRC were investigated using immunochemical staining on tissue microarrays. Afterward, survival analysis and stratification analysis were performed in stage II to explore the prognostic and predictive significance of *GADD45B*. Overexpressed *GADD45B* is associated with poorer prognosis for CRC patients both in overall survival (OS) (*p* < 0.001) and disease-free survival (DFS) (*p* = 0.001) based on the TCGA database. Analysis results according to the stage II CRC cohort and the liver metastatic CRC cohort revealed that *GADD45B* was gradually upregulated in normal mucosa including primary colorectal cancer (PCC). Colorectal liver metastases (CLM) tissues were arranged in order (normal tissue vs. PCC *p* = 0.005 and PCC vs. CLM *p* = 0.001). The low *GADD45B* group had a significantly longer five-year OS (*p* = 0.001) and progression-free survival (PFS) (*p* < 0.001) than the high *GADD45B* group for the stage II patients. The multivariate Cox regression analysis results proved that the expression level of *GADD45B* was an independent prognostic factor for stage II after radical surgery (OS: Hazard Ratio (HR) 0.479, [95% confidence interval (CI) 0.305–0.753] and PFS:HR 0.490, [95% CI 0.336–0.714]). In high *GADD45B* expression subgroup of stage II cohort, the patients who underwent adjuvant chemotherapy had longer PFS than those who did not (*p* = 0.008). High expression levels of *GADD45B* is an independent prognostic factor of decreased OS and PFS in stage II CRC patients. The stage II CRC patients with high *GADD45B* expression might benefit from adjuvant chemotherapy.

## 1. Introduction

Colorectal cancer (CRC) is one of the most common cancers worldwide. Colorectal cancer is threatening public health. In China, CRC new cases and specific death estimates were approximately 191,000 and 376,300 annually, respectively [1]. On account of the prevalence of early CRC screening, the health outcomes of patients diagnosed with stage I or stage II CRCs has improved. However, the treatment options of early stage CRC, especially stage II, remains controversial. About 20% of the CRC patients who underwent curative surgery alone will develop systemic metastasis [2]. In addition, the previous study revealed that chemotherapy could improve survival of stage II CRC patients but absolute improvement in survival was less than 5% [3]. Furthermore, the adverse events of adjuvant chemotherapy have an impact on the quality of life of patients [4]. Therefore, it is essential to identify valuable biomarkers to stratify the tumor progression risk after surgery in stage II CRC. In this way, patients who were predicted to have metastasis might get a benefit from the chemotherapy. Conversely, low-risk patients could avoid the associated adverse effects and save themselves the cost of chemotherapy.

The GADD45 family, which consists of *GADD45A* (*GADD45α*, *DDIT1*), *GADD45B* (*GADD45β*, *Myd118*), and *GADD45G* (*GADD45γ*, *cytokine-responsive 6*, *CR6*), participates in many cellular processes associated with cell growth regulation and the stress signaling pathway [5]. The proteins encoded by the GADD45 gene family are small (18kDa), conservative, and have homology and high acidity [6]. *GADD45B* shares the common functions of the GADD45 family, which is associated with DNA damage repair, cell growth, apoptosis, and anti-tumor immune responses [7,8]. Meanwhile, *GADD45B* might play critical roles in the tumorigenesis of human embryonic carcinoma [9], hepatocellular carcinoma [10], and pituitary gonadotrope tumors [11]. GADD45β is related to NF-kB, which is known to influence tumorigenesis, cancer cell survival, apoptosis, invasion, and metastasis and the GADD45 family are essential mediators of cell survival in cancer cells with implications for cancer chemotherapy and novel drug discovery [9]. A recent study implicated the carcinogenesis function and potential prognostic value of *GADD45B* for CRC [12]. However, the role of *GADD45B* expression in the prognostic value and chemotherapy-related predictive significance in stage II CRC remains uncertain, which should gain more attention.

In the present study, we first evaluated the expression patterns of *GADD45B* in CRC and assessed prognostic significance based on The Cancer Genomic Atlas (TCGA). We further investigated the *GADD45B* expression features of stage II and liver metastatic CRC using immunochemical staining according to the data in our cohort. Lastly, survival analysis and stratification analysis were performed in stage II to explore the prognostic and predictive value of *GADD45B*.

## 2. Materials and Methods

### 2.1. Patients

The specimens in this study were collected from the patients who underwent the surgical resection between 2006 and 2012 at the Cancer Institute & Hospital, Chinese Academy of Medical Sciences, Peking Union Medical College. All the diagnoses were confirmed according to the 7th edition American Journal of Critical Care (AJCC) TNM Classification. The inclusive criteria of stage II include the following: (A) AJCC pathology staging was stage II (T3-4N0M0); (B) no systemic or chemotherapy before the surgery; (C) the case can provide complete clinical information such as age, gender, tumor location, histology, differentiation, malignant tumors TNM classification, adjuvant therapy regime, follow-up information, and more. At the same time, we utilized the primary tumor and matched metastatic liver specimen to establish another simultaneous liver metastatic CRC (LMCRC) cohort. Totally, 306 cases of stage II CRC and 201 cases of simultaneous LMCRC were collected based on the inclusive criteria. All subjects gave their informed consent for inclusion before they participated in the study. The study was conducted in accordance with the Declaration of Helsinki, the Clinical Research Ethics Committee of Cancer Institute & Hospital, Chinese Academy of Medical Sciences approved this study (NCC2016JZ-06). All the patients were followed up every three months until 31 December 2017.

### 2.2. Genomic Analyses in the Public Dataset

To investigate the expression pattern and prognostic value of *GADD45B*, we analyzed colon cancer in 270 cases and rectal cancer in 92 cases provided by the TCGA project (Table S3). The Box Plots was generated to compare the *GADD45B* expression level between the tumor and normal tissues of CRC. The violin plots were created based on the patients’ pathological stages and *GADD45B* expression features. The Kaplan-Meier curves were plotted according to the expression value of *GADD45B*. The gene expression profiling interactive analysis (GEPIA) (http://gepia.cancer-pku.cn/index.html) is used for batch TCGA data processing and visualization in this study [13].

### 2.3. Tissue Microarray and Immunohistochemistry

**Figure 1:**
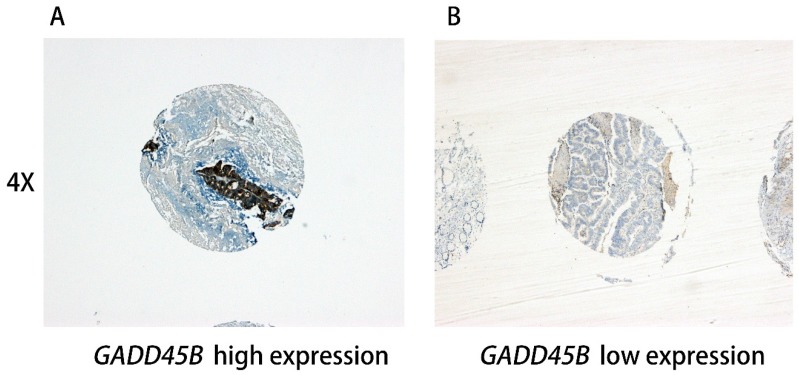

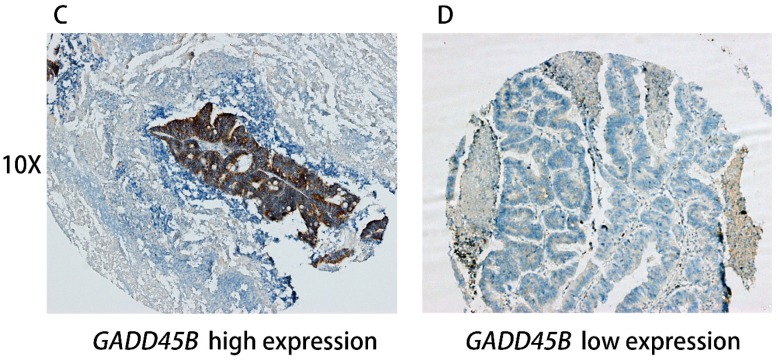
Representative Immunohistochemistry staining pictures of *GADD45B* expression in colorectal cancer tissues. Tissue high expression (4× for (**A**), 10× for (**C**)) and low expression (4× for (**B**), 10× for (**D**)) for the *GADD45B* protein are shown. Each of the punched samples is 1.0 mm in length in the tissue microarrays.

The stage II tissue microarrays (TMAs) consisted of the tumor sample and matched normal tissue from each patient and LMCRC TMAs consisted of the primary colorectal cancer (PCC), colorectal liver metastases (CLM), normal intestinal mucosa, and normal liver tissue from each patient. All tumor tissue microarrays were built after being verified by hematoxylin and eosin (H&E) staining. The punched sample measured 1.0 mm and was obtained from the center of the tumor. All the tissue microarrays were assessed by using immunohistochemical (IHC) staining and an anti-*GADD45B* antibody (ab105060; 1:500; Abcam; Cambridge, UK). The IHC staining is referenced to immunohistochemistry protocols published by the Abcam Company online [14]. The expression of the TMAs was scored by two independent pathologists in a blind study, according to the Staining Index (SI) that was previously used to assess the expression pattern of *GADD45B* [15,16]. The SI score was calculated by multiplying the staining intensity (0, negative; 1, weak; 2, moderate; 3, strong) and the percentage of positive stained cells (no staining, 0; 1–10%, 1; 11–50%, 2; 50–100%, 3). In this study, a moderate/strong cytoplasm staining of (SI = 3–9) was defined as positive staining while a weak or negative staining (SI = 0–2) was defined as negative staining. Representative staining of *GADD45B* in the specimens was illustrated in Figure 1. The positive rate refers to the proportion of *GADD45B* positive staining samples especially positive rate = positive samples/ (positive samples + negative samples).

### 2.4. Statistical Analysis

The student’s *t*-test was used to compare the difference of the data between two groups. The Chi-square test or Fisher exact test was used to evaluate categorical data. Survival curves were plotted according to the Kaplan-Meier method and the log-rank test was used to compare the OS (overall survival), DFS (disease-free survival), and PFS (progression-free survival) in the study cohorts. Univariate and multivariate analysis for CRC prognosis were undertaken using the Cox proportional hazards regression model. The calculations were performed with International Business Machines (IBM) SPSS Statistics version 24.0 software program and R version 3.3.3. A value of *p* < 0.05 was considered significant.

## 3. Results

### 3.1. High *GADD45B* Expression Is Associated with Poorer Overall Survival and Disease-Free Survival in Colorectal Cancer Patients from the Public Database

**Figure 2:**
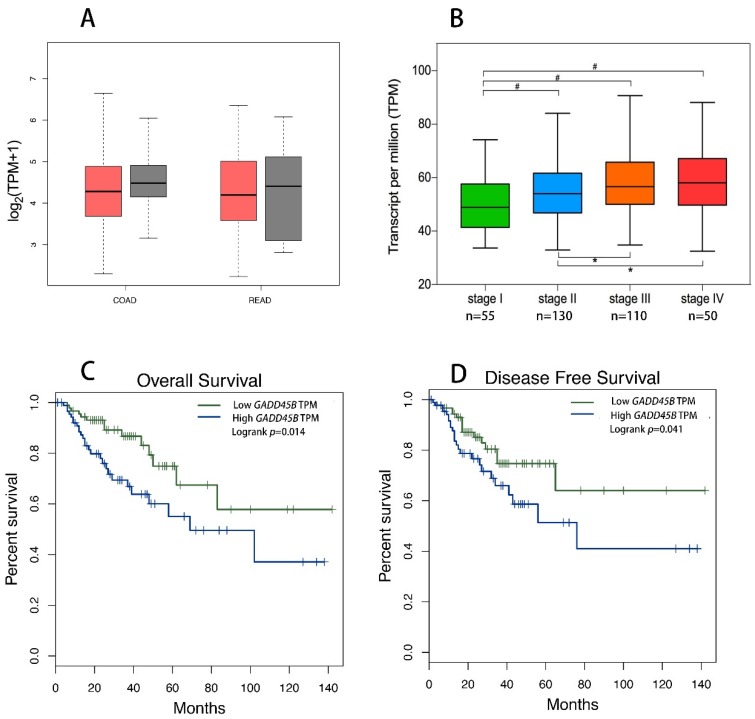
Expression of the *GADD45B* protein in CRC and Kaplan-Meier Curves of overall survival (OS) and disease-free survival (DFS) based on The Cancer Genomic Atlas (TCGA) database. The expression of *GADD45B* in colon cancer (COAD) and rectal cancer (READ) dataset was analyzed by gene expression profiling interactive analysis (GEPIA). The tumor was represented by a red color and the normal tissue was represented by a grey color (**A**). The GADD45 B expression box plots were generated based on CRC patient pathological major TNM staging (**B**). The most extreme value from bottom to top in the box plot represents the minimum value, the lower quartile, the median, the upper quartile, and the maximum value. The method for differential gene expression analysis is one-way Analysis of Variance (ANOVA) using the pathological stage as a variable for calculating differential expression. The *GADD45B* high expression group was associated with decreased OS (**C**) and DFS (**D**) in CRC according to the data from TCGA, which were calculated using a log-rank test. CRC: Colorectal cancer, TPM: Transcript per million, # represents *p*-value < 0.01, and * represents *p*-value < 0.05.

We analyzed the *GADD45B* expression level of the CRC cases from the TCGA database and there was no significant difference between the PCC and normal tissues (NT) in both the colon cancer (COAD) and rectal cancer (READ) dataset (Figure 2A). Meanwhile, the average expression level of *GADD45B* turned out to have a rising trend as the development of the TNM pathology stage (Figure 2B). As shown in the Kaplan-Meier curves (Figure 2C,D), overexpressed *GADD45B* is associated with a poorer prognosis for CRC patients both in OS and DFS (*p* = 0.014 for OS; *p* = 0.041 for DFS).

### 3.2. The *GADD45B* Expression Pattern in Stage II Colorectal Cancer and Liver Metastatic Colorectal Cancer Cohort

To validate the results analyzed by the public database, we detected the expression patterns of *GADD45B* in the stage II CRC cohort and the LMCRC cohort. In these two cohorts, 306 cases of stage II CRC and 201 cases of simultaneous LMCRC included respectively. All the IHC staining results have been listed in Table 1. In the stage II cohort, there was no significant difference regarding *GADD45B* protein expression in between the tumor tissue and the corresponding normal tissue (43.14% vs. 36.93%, *p* = 0.117). Based on the follow-up data, stage II was divided into the non-progression group (195 cases) and the progression group (111 cases) and 31 cases liver metastasis included in the latter group. Immunohistochemistry staining indicated that the progression group had a higher *GADD45B* expression positive rate than the non-progression group (50.45% vs. 38.97%, *p* = 0.004).

In the LMCRC cohort, *GADD45B* protein expression was detected in 105/201 (52.24%) of the PCC samples and 137/201 (68.16%) of the CLM samples (Table 1). At the same time, 77 cases (38.31%) of adjacent normal mucosa and 95 cases (47.26%) of the normal liver tissue showed staining-positive. Therefore, at protein levels, *GADD45B* was gradually upregulated in normal mucosa, PCC, and CLM tissues in order (normal tissue vs. PCC *p* = 0.005 and PCC vs. CLM *p* = 0.001).

We further compared the *GADD45B* expression of the primary tumor between stage II and LMCRC, and the results demonstrated that the latter was significantly upregulated (43.14% vs. 52.24%, *p* = 0.045). We also analyzed the *GADD45B* expression pattern in the primary tumor between stage II with liver metastasis after surgery and the simultaneous liver metastatic group. No significant difference was found (52.24% vs. 61.29%, *p* = 0.347).

### 3.3. The High GADD45B Expression Is Associated with Shorter Overall Survival and Progression-Free Survival in Stage II Colorectal Cancer

The association between the *GADD45B* expression of stage II primary and LMCRC patient pathologic characteristics is summarized in Table 2. However, the clinicopathologic information such as age, gender, tumor location, gross pathology type, differentiation grade, T stage, preoperative carcinoembryonic antigen (CEA) level, preoperative carbohydrate antigen 19-9(CA19-9) level, and microsatellite instability (MSI) status had no significant correlation with the *GADD45B* expression.

**Figure 3:**
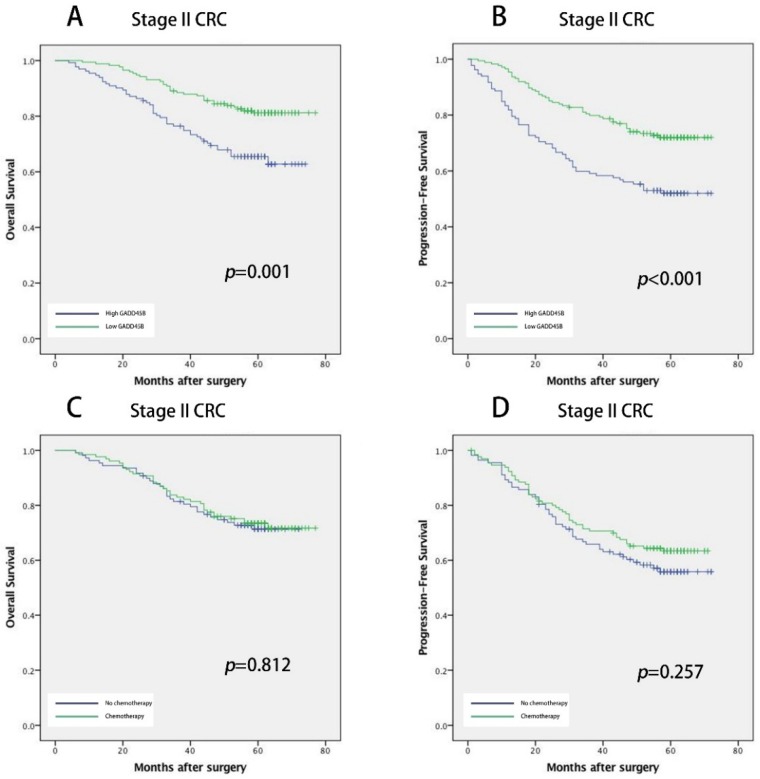

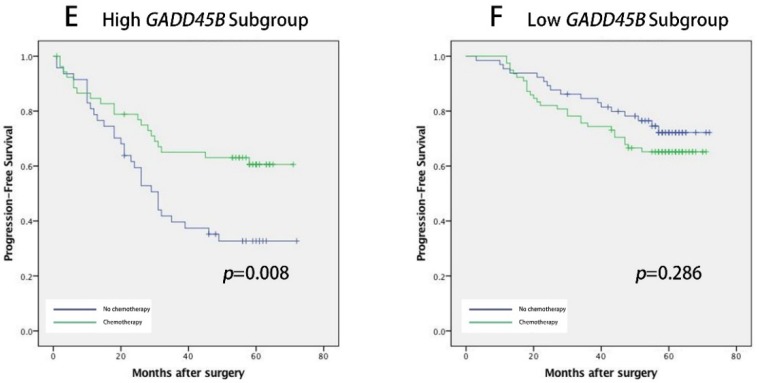
Prognostic power of *GADD45B* in stage II CRC cohort CRC cohort Kaplan-Meier analyses of overall survival and progression-free survival in patients with CRC based on the expression of *GADD45B*. Overall survival and progression-free survival (PFS) accord to *GADD45B* expression in stage II CRC (**A**,**B**). The relationship between *GADD45B* expression and PFS benefits from adjuvant chemotherapy in patients with stage II CRC. There was no significant difference in OS and PFS between the patients who received chemotherapy and the patients who never received the adjuvant therapy after surgery (**C**,**D**). Treatment with 5-Fluorouracil-based (5-FU-based) chemotherapy was associated with a higher rate of PFS in the *GADD45B* high group (**E**) but not in the *GADD45B* low group (**F**).

To identify the prognosis value of *GADD45B* expression in CRC, the Kaplan Meier survival analyses were conducted in stage II. The median follow-up was more than 59 months and 78 cases of death, and 111 relapsed patients included. Kaplan Meier analysis revealed that in stage II patients, the low *GADD45B* group had a significantly longer five-year OS and PFS than the high *GADD45B* group (*p* = 0.001 for OS and *p* < 0.001 for PFS; Figure 3A,B).

### 3.4. *GADD45B* Expression Is an Independent Prognostic Factor in Stage II

Univariate Cox regression analysis confirmed that high *GADD45B* was associated with a worse prognosis significance for OS (HR 0.459, [95% CI 0.292–0.721]) and PFS (HR 0.480, [95% CI 0.330–0.699], Table 3 and Table 4, Tables S1 and S2). In the multivariate Cox regression analysis, high *GADD45B* expression was also significantly associated with the poorer rate of OS (HR 0.479, [95% CI 0.305–0.753]) and PFS (HR 0.490, [95% CI 0.336–0.714], Table 3 and Table 4, Tables S1 and S2). The results proved that low-level expression of *GADD45B* was an independent prognostic factor for stage II after radical surgery. Furthermore, neurological involvement was also a significant independent prognostic factor for PFS in the stage II cohort.

### 3.5. Expression of GADD45B Could Predict the Benefit from the Adjuvant Chemotherapy in Stage II Colorectal Cancer

Next, we investigated the potential role of *GADD45B* as a predictor of effectiveness of chemotherapy in stage II. In this cohort, 131 patients received the 5-Fluorouracil-based (5-FU-based) adjuvant chemotherapy, 63 lower rectal cancer patients underwent radiotherapy (50 gray (Gy)), and 112 patients didn’t take any adjuvant therapy after surgery. There appeared to be no significant difference in OS and PFS between the patients who received chemotherapy and the patients who never received the adjuvant treatment after surgery (*p* = 0.812 for OS and *p* = 0.257 for PFS; Figure 3C,D). To assess the predictive value of *GADD45B* for an adjuvant chemotherapy benefit, we compared the PFS of stage II CRC patients who underwent adjuvant chemotherapy or not in the subgroup after being stratified by *GADD45B* expression. In the high *GADD45B* expression subgroup, chemotherapy was associated with a higher rate of PFS (*p* = 0.008; Figure 3E), but this relationship did not exist in the subgroup of low *GADD45B* expression (*p* = 0.286, Figure 3F). The test of interaction results indicated that the benefit associated with adjuvant chemotherapy in the high *GADD45B* expression group was superior to the low *GADD45B* expression group (*p* = 0.027).

## 4. Discussion

In the present study, we evaluated the *GADD45B* expression pattern of different CRC development stages and prognostic values of *GADD45B* in stage II CRC. We first explored expression features of *GADD45B* in CRC based on the TCGA database and two CRC cohorts. As illustrated in the violin figure plotted by GEPIA, the expression level of *GADD45B* increased due to the development of the TNM stage. The results analyzed by our cohorts also indicated that the positive rate of *GADD45B* expression was rising in the primary tumor of stage II, PCC, and CLM of liver metastasis in order. Moreover, the IHC positive rate of *GADD45B* of the stage II progression group was higher than the non-progression group, but it turned out to have no significant difference in the primary tumor between stage II with liver metastasis after surgery and the simultaneous liver metastatic group. An associated study indicated that *GADD45B* contributed to tumor progression rather than the initiation in hepatocellular carcinoma and ovarian cancer [17]. Therefore, we infer that *GADD45B* may also be regarded as a potential tumor progression predictive marker in CRC.

When we made a survival analysis based on the TCGA data, the results suggested that the expression of *GADD45B* was significantly associated with the prognosis of CRC both in OS and PFS. Subsequently, the survival analysis was performed in the stage II cohort to validate the prognostic significance in this stage. It was shown that high expression of *GADD45B* was an independent prognostic factor of decreased OS and PFS accounting for stage II CRC. It was found that *GADD45B* had a predictive value to estimate the benefit from 5-FU-based chemotherapy in stage II. Additionally, in the previous studies, *GADD45B* was demonstrated to induce apoptosis and repair DNA damage in CRC cell lines, which plays a role in the tumor suppressor gene. This result seemed to be inconsistent with our study. However, a study published by Wang et al. [12] implicated that *GADD45B* might lose its normal functions and promote carcinogenesis and tumor progression in CRC tissues. At the same time, with the accumulation of DNA damage in tumor progression, the *GADD45B* expression level will improve feedback. 

*GADD45B*eta is a stress-activated protein that plays a vital role in regulating apoptosis, proliferation, and DNA repair. It has been demonstrated to be an indicator for predicting clinical outcomes of gastric cancer [18], ovarian cancer [19], and glioma [20] according to the previous studies. Verzella et al. found that elevated *GADD45B* expression correlated with rapid disease progression in 13 of the top 15 solid cancers for mortality and the patient cohorts expressing high *GADD45B* levels exhibited significantly shorter recurrence-free survival and OS than the corresponding cohorts, which expressed low *GADD45B* messenger-RNA (mRNA) levels [16]. Even though a previous study has revealed the prognostic value of *GADD45B* in CRC, its validation cohort only included 152 cases with stage II–III [12]. Our study was performed to better understand the adjuvant chemotherapy indication-related problem for stage II patients with better clinical value. The results suggested that the benefit associated with adjuvant chemotherapy in the high *GADD45B* expression group was superior to the low *GADD45B* expression group. This result would provide stage II patients with a better selection for adjuvant chemotherapy to promote clinical outcomes and decrease overtreatment and cost. It was found that the stage II patients with low expression GAD45BB could appropriately change their chemotherapy regime or simply “look and wait.” Moreover, if we want to translate the finding into a better treatment strategy, more cohorts and larger size cases are needed and a related prospective study is also necessary.

This study has some limitations. First, our validation cohorts only consist of stage II and liver metastatic CRC patients and lack the other stages. Therefore, the next step should be to complete the CRC cohort establishment of stage I, stage III, and even precancerous stages to further vindicate the *GADD45B* expression characteristics in the development of CRC. In addition, all the cases in the cohort were collected from one single institution, which means further validations form multicenter and larger sample size cohorts are needed.

Currently, checkpoint inhibitor treatment is a major problem in tumor immunotherapy. Although immunotherapy has not been carried out in CRC widespread, it was shown that *GADD45B* could modulate innate immune checkpoint functions, which are amenable to therapeutic intervention to reprogram tumor-associated macrophages and ultimately overcome tumor microenvironments dependent on immunosuppression [17,21]. Moreover, as mentioned in the discussion above, inhibition of the *GADD45B* expression leads to suppression of proliferation and further prohibits tumor progression. Collectively, *GADD45B* is not only a prognostic factor in CRC but also a promising potential treatment target in the future.

## 5. Conclusions

The results suggest that a high expression level of *GADD45B* is an independent prognostic factor of decreased OS and PFS in stage II CRC patients. The stage II CRC patients with high *GADD45B* expression may benefit from adjuvant chemotherapy.

## Figures and Tables

**Table 1:** The GADD45B expression pattern in different samples by immunohistochemical (IHC) staining.

	Sample	*GADD45B*-Positive	*GADD45B*-Negative	Positive Rate
Stage II	Tumor	132	174	43.14%
	Normal Tissue	113	193	36.93%
	Progression	60	51	50.45%
	Non-progression	72	123	38.97%
	Recurrence of Liver Metastasis after Surgery	19	12	61.29%
LMCRC	Primary Tumor	105	96	52.24%
	Normal Colorectal Mucosa	77	124	38.31%
	Liver Metastasis	137	64	68.16%
	Normal Liver Tissue	95	106	47.26%

Stage II: Stage II colorectal cancer, LMCRC: Liver metastatic colorectal cancer, Progression: Tumour recurrence after surgery in 5 years, Non-Progression: No recurrence signs after surgery in 5 years.

**Table 2:** Correlation between the intensity of *GADD45B* expression and the clinical profiles of stage II colorectal cancer.

Factor	Total	*GADD45B* HIGH (%)	*GADD45B* LOW (%)	*p* Value
**Age (years)**				
<65	216	89 (41.2)	127 (58.8)	0.290
≥65	90	43 (47.8)	47 (52.2)	
**Gender**				
Male	183	78 (42.6)	105 (57.4)	0.825
Female	123	54 (43.9)	69 (56.1)	
**Tumor Location**				
Colon	143	59 (72.4)	84 (27.6)	0.836
Rectum	163	73 (71.3)	90 (28.7)	
**Gross Pathological Type**				
Prominence	171	64 (41.3)	107 (58.7)	0.534
Ulceration & Infiltration	135	68 (44.8)	67 (55.2)	
**Grade**				
High	34	18 (52.9)	16 (47.1)	0.409
Middle	240	102 (42.5)	138 (57.5)	
Low	32	12 (37.5)	20 (62.5)	
**T Stage**				
T3	286	125 (43.7)	161 (56.3)	0.175
T4	20	17 (56.7)	13 (43.3)	
**Adjuvant Therapy**				
Chemotherapy	131	53 (40.5)	78 (59.5)	0.377
Radiotherapy	63	32 (50.8)	31 (49.2)	
No	112	47 (42.0)	65 (58.0)	
**Preoperative CEA Level** **(ng/mL** **)**				
≤5	244	101 (41.4)	143 (58.6)	0.222
>5	62	31 (50.0)	31 (50.0)	
**Preoperative CA19-9 Level** **(U/mL** **)**				
≤37	282	119 (42.2)	163 (57.8)	0.182
>37	23	13 (56.5)	10 (43.5)	
**MSI**				
MSS	268	120 (44.8)	148 (55.2)	0.124
MSI	38	12 (31.6)	26 (68.4)	

CEA: Carcinoembryonic antigen; CA19-9: Carbohydrate antigen 19-9; MSI: Microsatellite instability; MSS: Microsatellite stable; T stage: Tumour stage.

**Table 3:** Cox analyses of potential prognostic factors for overall survival in the stage II CRC cohort.

Factor	Comparison	Univariate Analysis			Multivariate Analysis		
		HR	95% CI	*p* Value	HR	95% CI	*p* Value
**MSI Status**	MSI vs. MSS	0.343	0.126–0.939	0.037	0.379	0.138–1.039	0.059
***GADD45B* Expression**	LOW vs. HIGH	0.459	0.292–0.721	0.001	0.479	0.305–0.753	0.001

HR: Hazard ratio; CI: confidence interval.

**Table 4:** Cox analyses of potential prognostic factors for progression-free survival in the stage II CRC cohort.

Factor	Comparison	Univariate Analysis			Multivariate Analysis		
		HR	95% CI	*p* Value	HR	95% CI	*p* Value
**Neurological Involvement**	Present vs. Absent	2.222	1.191–4.145	0.012	2.066	1.107–3.858	0.023
***GADD45B* Expression**	LOW vs. HIGH	0.480	0.330–0.699	0.0001	0.490	0.336–0.714	0.0002

## Supplementary Materials

The following are available online at http://www.mdpi.com/2073-4425/9/7/361/s1. Table S1: Cox analyses of potential prognostic factors for overall survival in the stage II CRC cohort, Table S2: Cox analyses of potential prognostic factors for progression-free survival in the stage II CRC cohort; Table S3: Colon cancer and rectal cancer samples provided by the TCGA project.
