# Supplementary material for: GADD45B as a Prognostic and Predictive Biomarker in Stage II Colorectal Cancer
Source: Genes (Basel). 2018 Jul 19;9(7):361. doi: 10.3390/genes9070361 (PMC6071283; doi:10.3390/genes9070361)
Supplement: Supplementary file 1 [file genes-09-00361-s001.zip › Supplementary materials.docx]

**Supplementary Files**

| **Table S1.**  **Cox analyses of potential prognostic factors for overall survival in the stage II CRC cohort** | | | | | | | | |
| --- | --- | --- | --- | --- | --- | --- | --- | --- |
| **Factor** | Comparison | Univariate Analysis | | |  | Multivariate Analysis | | |
|  |  | HR | 95%CI | *p* value |  | HR | 95%CI | *p* value |
| **Age(years)** | ＜65 vs. ≥65 | 1.502 | 0.949-2.379 | 0.083 |  |  |  |  |
| **Gender** | Female vs. Male | 0.901 | 0.721-1.125 | 0.356 |  |  |  |  |
| **Tumor Location** | Colon Cancer vs.  Rectal Cancer | 0.940 | 0.752-1.174 | 0.585 |  |  |  |  |
| **Gross Pathological Type** | Prominence.  vs.  Ulceration &Infiltration | 1.312 | 0.837-2.057 | 0.237 |  |  |  |  |
| **T stage** | T3 vs. T4 | 1.493 | 0.687-3.247 | 0.312 |  |  |  |  |
| **Grade** | High& Middle vs. Low | 1.013 | 0.548-1.874 | 0.967 |  |  |  |  |
| **Neurological Involvement** | Present vs. Absent | 1.731 | 0.796-3.763 | 0.166 |  |  |  |  |
| **Vascular Invasion** | Present vs. Absent | 0.553 | 0.206-1.484 | 0.240 |  |  |  |  |
| **Adjuvant Therapy** | Yes vs. No | 0.821 | 0.528-1.278 | 0.383 |  |  |  |  |
| **Chemotherapy** | Yes vs. No | 1.045 | 0.648-1.688 | 0.856 |  |  |  |  |
| **CEA level.**  **（ng/ml）** | ≤5vs.＞5 | 0.925 | 0.534-1.602 | 0.780 |  |  |  |  |
| **CA19-9 level.**  **（U/ml）** | ≤37vs.＞37 | 0.675 | 0.247-1.847 | 0.444 |  |  |  |  |
| **MSI status** | MSI vs. MSS | 0.343 | 0.126-0.939 | 0.037 |  | 0.379 | 0.138-1.039 | 0.059 |
| ***GADD45B* expression** | LOW vs. HIGH | 0.459 | 0.292-0.721 | 0.001 |  | 0.479 | 0.305-0.753 | 0.001 |

| **Table S2**  **Cox analyses of potential prognostic factors for progression-free survival in the stage II CRC cohort** | | | | | | | | | |
| --- | --- | --- | --- | --- | --- | --- | --- | --- | --- |
| **Factor** | Comparison | Univariate Analysis | | |  | Multivariate Analysis | | | |
|  |  | HR | 95%CI | *p* value | |  | HR | 95%CI | *p* value |
| **Age(years)** | ＜65 vs. ≥65 | 1.163 | 0.781-1.730 | 0.458 | |  |  |  |  |
| **Gender** | Female vs. Male | 0.926 | 0.635-1.351 | 0.690 | |  |  |  |  |
| **Tumor Location** | Colon Cancer vs.  Rectal Cancer | 0.966 | 0.802-1.164 | 0.717 | |  |  |  |  |
| **Gross Pathological Type** | Prominence  vs.  Ulceration& Infiltration | 1.194 | 0.820-1.738 | 0.356 | |  |  |  |  |
| **T Stage** | T3 vs. T4 | 1.337 | 0.676-2.644 | 0.404 | |  |  |  |  |
| **Grade** | High& Middle vs. Low | 1.078 | 0.643-1.806 | 0.777 | |  |  |  |  |
| **Neurological Involvement** | Present vs. Absent | 2.222 | 1.191-4.145 | 0.012 | |  | 2.066 | 1.107-3.858 | 0.023 |
| **Vascular Invasion** | Present vs. Absent | 0.948 | 0.575-1.562 | 0.834 | |  |  |  |  |
| **Adjuvant Therapy** | Yes vs. No | 0.908 | 0.626-1.318 | 0.613 | |  |  |  |  |
| **Chemotherapy** | Yes vs. No | 1.092 | 0.723-1.650 | 0.675 | |  |  |  |  |
| **CEA level**  **（ng/ml）** | ≤5 vs.＞5 | 1.088 | 0.697-1.698 | 0.711 | |  |  |  |  |
| **CA19-9 level**  **（U/ml）** | ≤37 vs.＞37 | 0.720 | 0.316-1.640 | 0.435 | |  |  |  |  |
| **MSI status** | MSI vs. MSS | 0.625 | 0.326-1.197 | 0.157 | |  |  |  |  |
| ***GADD45B* expression** | LOW vs. HIGH | 0.480 | 0.330-0.699 | 0.0001 | |  | 0.490 | 0.336-0.714 | 0.0002 |
