# Supplementary material for: GADD45B as a Prognostic and Predictive Biomarker in Stage II Colorectal Cancer
Source: Genes (Basel). 2018 Jul 19;9(7):361. doi: 10.3390/genes9070361 (PMC6071283; doi:10.3390/genes9070361)
Supplement: Supplementary file 1 [file genes-09-00361-s001.zip › Supplementary materials.pdf]

genes

MDPI

Supplementary Files

Table S1.

Cox analyses of potential prognostic factors for overall survival in the stage II CRC cohort

Factor	Comparison	Univariate Analysis			Multivariate Analysis		
		HR	95%CI	*p* value	HR	95%CI	*p* value
**Age(years)**	＜65 vs. ≥65	1.502	0.949-2.379	0.083			
**Gender**	Female vs. Male	0.901	0.721-1.125	0.356			
**Tumor Location**	Colon Cancer vs. Rectal Cancer	0.940	0.752-1.174	0.585			
**Gross Pathological Type**	Prominence. vs. Ulceration &Infiltration	1.312	0.837-2.057	0.237			
**T stage**	T3 vs. T4	1.493	0.687-3.247	0.312			
**Grade**	High& Middle vs. Low	1.013	0.548-1.874	0.967			
**Neurological Involvement**	Present vs. Absent	1.731	0.796-3.763	0.166			
**Vascular Invasion**	Present vs. Absent	0.553	0.206-1.484	0.240			
**Adjuvant Therapy**	Yes vs. No	0.821	0.528-1.278	0.383			
**Chemotherapy**	Yes vs. No	1.045	0.648-1.688	0.856			
**CEA level.（ng/ml）**	≤5vs.＞5	0.925	0.534-1.602	0.780			
**CA19-9 level.（U/ml）**	≤37vs.＞37	0.675	0.247-1.847	0.444			
**MSI status**	MSI vs. MSS	0.343	0.126-0.939	0.037	0.379	0.138-1.039	0.059
***GADD45B* expression**	LOW vs. HIGH	0.459	0.292-0.721	0.001	0.479	0.305-0.753	0.001

Table S2

Cox analyses of potential prognostic factors for progression-free survival in the stage II CRC cohort

**Factor**	Comparison	Univariate Analysis			Multivariate Analysis		
		HR	95%CI	*p* value	HR	95%CI	*p* value
**Age(years)**	＜65 vs. ≥65	1.163	0.781-1.730	0.458			
**Gender**	Female vs. Male	0.926	0.635-1.351	0.690			
**Tumor Location**	Colon Cancer vs. Rectal Cancer	0.966	0.802-1.164	0.717			
**Gross Pathological Type**	Prominence vs. Ulceration& Infiltration	1.194	0.820-1.738	0.356			
**T Stage**	T3 vs. T4	1.337	0.676-2.644	0.404			
**Grade**	High& Middle vs. Low	1.078	0.643-1.806	0.777			
**Neurological Involvement**	Present vs. Absent	2.222	1.191-4.145	0.012	2.066	1.107-3.858	0.023
**Vascular Invasion**	Present vs. Absent	0.948	0.575-1.562	0.834			
**Adjuvant Therapy**	Yes vs. No	0.908	0.626-1.318	0.613			
**Chemotherapy**	Yes vs. No	1.092	0.723-1.650	0.675			
**CEA level （ng/ml）**	≤5 vs.＞5	1.088	0.697-1.698	0.711			
**CA19-9 level （U/ml）**	≤37 vs.＞37	0.720	0.316-1.640	0.435			
**MSI status**	MSI vs. MSS	0.625	0.326-1.197	0.157			
***GADD45B* expression**	LOW vs. HIGH	0.480	0.330-0.699	0.0001	0.490	0.336-0.714	0.0002
